# Analysis of SARS-CoV-2 PCR and Chest CT Findings at a Single Facility in Tokyo (Machida Municipal Hospital)

**DOI:** 10.1155/2021/6680232

**Published:** 2021-07-26

**Authors:** Taisuke Kazuyori, Akihito Sato, Yasuhiro Morimoto, Kazuyoshi Kuwano

**Affiliations:** ^1^Machida Municipal Hospital, Department of Respiratory Medicine, Tokyo 194-0023, Japan; ^2^The Jikei University School of Medicine, Division of Respiratory Medicine, Tokyo 105-8461, Japan

## Abstract

**Introduction:**

The SARS-CoV-2 reverse transcription polymerase chain reaction (RT-PCR) test is useful for diagnosing COVID-19, and the RT-PCR positive rate is an important indicator for estimating the incidence rate and number of infections. COVID-19 pneumonia is also associated with characteristic findings on chest CT, which can aid in diagnosis.

**Methods:**

We retrospectively evaluated patient background characteristics, the number of cases, the positivity rate, and chest CT findings for positive and negative cases in 672 patients who underwent RT-PCR for suspected COVID-19 at our hospital between April 3 and August 28, 2020. In addition, we compared trends in the positive rates at approximately weekly intervals with trends in the number of new infections in Machida City, Tokyo.

**Results:**

The study included 323 men and 349 women, with a median age of 46 years (range: 1 month–100 years). RT-PCR findings were positive in 37 cases, and the positive rate was 5.51%. Trends in the positive rate at our hospital and the number of new COVID-19 cases in the city were similar during the study period. Among patients with positive results, 15 (40.5%) had chest CT findings, and 14 had bilateral homogeneous GGOs. Among patients with negative results, 190 had chest CT findings at the time of examination, and 150 were diagnosed with bacterial pneumonia or bronchitis, with main findings consisting of consolidations and centrilobular opacities. Only 11 of these patients exhibited bilateral homogeneous GGOs.

**Conclusion:**

Bilateral homogeneous GGOs are characteristic of COVID-19 pneumonia and may aid in the diagnosis of COVID-19.

## 1. Introduction

In December 2019, coronavirus disease 2019 (COVID-19) caused by severe acute respiratory syndrome coronavirus 2 (SARS-CoV-2) began to spread from Wuhan, China, to countries worldwide, including Japan. By August 2020, the number of COVID-19 cases exceeded 25 million, and more than 850,000 had people died [1]. In Japan, a state of emergency against the spread of COVID-19 was declared from April 7 to May 25, 2020. Despite a decrease in the number of COVID-19 infections after lifting the state of emergency, the number of cases increased again in late June and peaked in early August, gradually decreasing thereafter.

Although the SARS-CoV-2 reverse transcriptase polymerase chain reaction (RT-PCR) test is a highly specific test and is used for definitive diagnosis, it is unsuitable for use as a screening test owing to the long time required to obtain results, its high cost, and inadequate sensitivity. While medical institutions in Japan initially sent RT-PCR tests to public health centers for analysis, they can now request test results from private laboratories. Indeed, our hospital has requested RT-PCR from private laboratories since April 3.

In general, the positive rate obtained from tests performed to diagnose certain diseases is used as an indicator to estimate patient morbidity. In the case of an extremely specific test, morbidity is calculated based on the positive rate and sensitivity of the test: assuming that sensitivity is a constant, morbidity can be derived from the positive rate. Since this is also true for RT-PCR tests for COVID-19, the RT-PCR positive rate is an important indicator for estimating changes in morbidity and the number of infections. Our institution is a public hospital in Machida City, Tokyo, and most patients are local residents. Thus, one can assume that the positive test rate at our hospital accurately reflects the COVID-19 situation in the city.

COVID-19 is also frequently associated with pneumonia, and chest computed tomography (CT) can aid in diagnosis. Characteristic findings of COVID-19 pneumonia used for diagnosis include bilateral, nonregional ground glass opacities (GGOs) and focal consolidation on chest CT [2–13]. However, it remains unclear whether there is a difference in chest CT findings between patients with positive and negative RT-PCR results among those with suspected COVID-19 treated at a community hospital.

Therefore, in the present study, we calculated the number of positive cases and the positive rate for COVID-19 RT-PCR tests performed at our hospital and compared chest CT findings between patients with positive and negative results.

## 2. Materials and Methods

We analyzed data related to sex, age, and presence or absence of abnormal findings on chest radiographs for 672 patients who underwent RT-PCR (LightMix® Modular SARS-CoV (COVID19) E-gene or Cobas® SARS-CoV-2, Roche Diagnostics K.K) at our hospital for suspected COVID-19 from April 3 to August 28, 2020. In addition to the overall RT-PCR positive rate, we compared trends in positive rates at approximately weekly intervals with trends in the number of new infections in Machida City, Tokyo. The number of new infections in Machida City was based on data published on the Machida City government website [14]. Furthermore, for cases with active findings on chest CT, we compared background factors (age, sex, smoking history, severity, CT findings, outcome, and comorbidities) between RT-PCR-positive and RT-PCR-negative cases. We also compared the findings that were characteristic of RT-PCR-positive cases and examined whether these findings could be confirmed in RT-PCR-negative cases. EZR software (ver. 1.42) was used for statistical analysis [15]. This study was approved by the institutional ethics committee of our hospital. The study was conducted in accordance with the Declaration of Helsinki and the Ethical Guidelines for Medical and Health Research Involving Human Subjects.

## 3. Results

**Figure 1:**
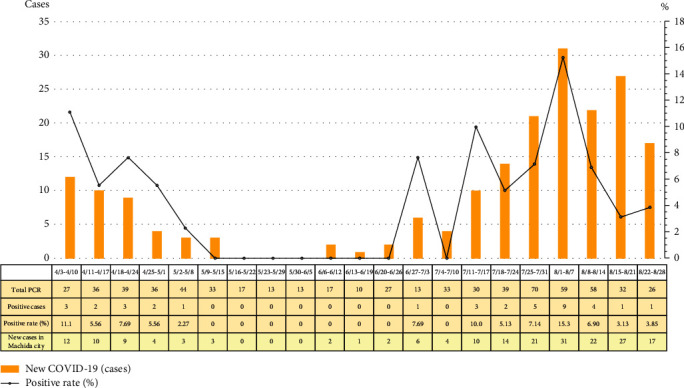
Trends in the reverse transcription polymerase chain reaction (RT-PCR) positive rate and number of new COVID-19 cases in Machida City.

The background characteristics of the 672 patients (323 men, 349 women; median age: 46 years (range: 1 month-100 years)) who underwent RT-PCR are shown in Table 1. The number of positive cases was 37, and the positive rate was 5.5%. Trends in the RT-PCR positivity rate for our hospital and the number of new cases of COVID-19 in Machida City are illustrated in Figure 1. The figure shows that the two trends were almost identical.

**Figure 2:**
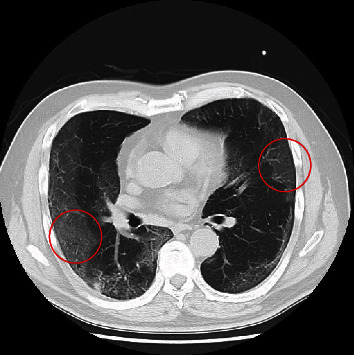
Bilateral homogenous ground-glass opacities (GGOs) in a patient with positive reverse transcription polymerase chain reaction (RT-PCR) findings (Case 2). Chest CT shows bilateral homogenous concentrations of GGOs (inside the circle) distributed in the subpleural region.

Chest CT findings were observed in 205 cases. Among patients with positive RT-PCR results, 15 exhibited some chest CT findings, and their background characteristics are shown in Table 2. Disease severity was mild in six cases (no oxygen therapy required), moderate in six cases (oxygen therapy required), and severe in three cases (respiratory failure requiring ventilatory therapy). One patient died due to severe complications of COVID-19. Bilateral homogeneous GGOs (Figure 2) were observed on chest CT in 14 cases (93%). A comparison of initial CT findings between mild-to-moderate and severe cases (including fatal cases) revealed that severe cases were associated with more extensive GGOs and consolidation than mild-to-moderate cases (mild to moderate: 6 [50%]; severe: 3 [100%]).

Table 3 shows diagnoses among RT-PCR-negative cases: 150 of 190 cases had bacterial pneumonia/bronchitis, 27 cases had heart failure, and 20 cases had interstitial lung disease (ILD). Only 11 of the RT-PCR-negative cases had bilateral homogeneous GGOs, which were significantly more common among RT-PCR-positive cases (Fisher's exact test, *P* < .01).

Among patients with bacterial pneumonia/bronchitis, heart failure, and ILD, bilateral homogeneous GGOs were found in one, two, and seven cases, respectively. Significantly more bilateral homogeneous GGOs were observed in patients with ILD than in those with bacterial pneumonia/bronchiolitis and heart failure (Fisher's exact test, *P* < .05). However, COVID-19 was associated with significantly more bilateral homogenous GGOs than ILD (Fisher's exact test, *P* < .01).

## 4. Discussion

In this study, we compared trends in the RT-PCR positive rate with the number of new COVID-19 infections in Machida City (e.g., the administrative district to which our hospital belongs). Our findings indicated that the two trends were nearly identical. In our hospital, RT-PCR was performed in cases of suspected COVID-19, and we did not perform RT-PCR for randomly selected residents to assess the prevalence of COVID-19 in the community, in accordance with the examination policy in Japan at that time. Since there were no nosocomial infections at our hospital or outbreaks in neighboring areas, otherwise known as “clusters,” we speculate that the RT-PCR results at our hospital and the number of new cases in Machida City did not differ significantly.

In addition, half of patients with positive RT-PCR results had findings on chest CT, most of which were bilateral homogeneous GGOs. Several previous studies have reported that early COVID-19 is associated with bilateral GGOs on chest CT [10–12]. According to two meta-analyses, other typical imaging findings include interlobular septal thickening, adjacent pleural thickening, reticulation, and air bronchogram [2, 13]. The bilateral homogeneous GGOs observed among our patients were similar to those found in the early stages of COVID-19 in a previous study [12]. Such findings are rarely observed in patients with bacterial pneumonia and bronchitis diagnosed at the same time and may be important in differentiating COVID-19 from other forms of pneumonia. However, given that ILDs—especially drug-induced pneumonia, cryptogenic organizing pneumonia, and eosinophilic pneumonia—are associated with findings that are relatively similar to bilateral homogeneous GGOs in combination with consolidations [16–18], clinicians should carefully conduct medical interviews and examine other laboratory findings to differentiate ILD from COVID-19.

Machida City is located in the southwestern part of the Tama region of Tokyo and has a population of approximately 430,000. The Machida Municipal Hospital is the only public hospital in Machida City and is a regional center of medical care, including acute care. Since many emergency patients visit the hospital, it is very important to identify COVID-19 among them, but differential diagnosis is often difficult. In this study, the number of new COVID-19 cases in Machida City and the rate of positive PCR tests in our hospital were in general agreement. The PCR positivity rate can be used to predict the prevalence of COVID-19 in the region, and the system for treating COVID-19 can be adjusted based on its prevalence. In addition, because bilateral homogeneous GGOs are characteristic of COVID-19, they may aid in differentiating COVID-19 from other forms of pneumonia. Thus, our results have important implications for both COVID-19 treatment and regional medicine.

Our study had some limitations. First, it was difficult to assess the daily positive rate given the small number of cases, and the study was conducted at a single institution and may not necessarily reflect the situation in a wider area such as Machida City. In addition, because this study was retrospective, further prospective studies are required to evaluate the usefulness of bilateral homogeneous GGOs for the diagnosis of COVID-19. However, by dividing the testing period and new cases into intervals of approximately 1 week, we were able to assess trends in the positive rates and the number of new cases in Machida City. Our study may be useful for many institutions that must balance COVID-19 care and conventional medical practice.

## 5. Conclusions

Our analysis indicated that the RT-PCR positive rate in our hospital and the number of new COVID-19 cases in Machida City tended to be similar. Given that bilateral homogeneous GGOs are characteristic of COVID-19 pneumonia, these CT findings may aid in the diagnosis of COVID-19.

## Figures and Tables

**Table 1:** Background characteristics.

Total *n* = 672	
Age (%)	
0-10	75 (11.1)
10-20	29 (4.3)
20-30	103 (15.3)
30-40	73 (10.9)
40-50	94 (14.0)
50-60	60 (8.9)
60-70	48 (7.1)
70-80	72 (10.7)
80-90	94 (14.0)
90-	24 (3.6)
Median: 46 (1 month, 100 y)	
Male (%)	323 (48.1)
Female (%)	349 (51.9)

Positive (%)	37 (5.5)
Negative (%)	635 (94.5)

CT findings (%)	205 (30.5)

2020/4/3-2020/6/26 (%)	312 (46.4)
2020/6/27-2020/8/28 (%)	360 (53.6)

**Table 2:** Background characteristics of patients with positive COVID-19 RT-PCR results with chest findings on CT.

Case No.	Age	Sex	Smoking history	Severity^∗^	Bilateral homogeneous GGOs on chest CT	Outcome	Comorbidities
1	55	Male	<20 pack-years	Moderate	+	Recovered	None
2	60	Male	≥20 pack-years	Severe	+	Recovered	Diabetes mellitus, renal cancer
3	61	Male	≥20 pack-years	Moderate	+	Recovered	None
4	59	Male	Never	Moderate	+	Recovered	Hypertension, diabetes mellitus, cardiac disease
5	67	Male	<20 pack-years	Moderate	+	Recovered	None
6	57	Female	≥20 pack-years	Severe	+	Recovered	Chronic renal failure (hemodialysis), polycystic kidney disease
7	21	Female	Never	Mild	+	Recovered	None
8	57	Male	Never	Mild	+	Recovered	Ulcerative colitis
9	20	Female	Unknown	Mild	-	Recovered	None
10	86	Male	Never	Moderate	+	Recovered	Hypertension
11	85	Male	<20 pack-years	Severe	+	Death	Hypertension, diabetes mellitus, cardiac disease, aspiration pneumonia
12	47	Male	<20 pack-years	Moderate	+	Recovered	Diabetes mellitus, sleep apnea syndrome
13	57	Female	Never	Mild	+	Recovered	Hypertension
14	31	Male	<20 pack-years	Mild	+	Recovered	None
15	45	Female	Never	Mild	+	Recovered	Cough variant asthma

^∗^Mild: oxygen therapy not required; moderate: oxygen therapy required; severe: respiratory failure requiring ventilatory therapy.

**Table 3:** Diagnoses and frequency of bilateral homogeneous ground-glass opacities (GGOs) on chest CT in reverse transcription polymerase chain reaction- (RT-PCR-) negative cases.

Diagnosis^∗^	All cases *n* = 190	Bilateral homogeneous GGOs *n* = 11
Pneumonia/bronchitis	150	1
Heart failure	27	2
Interstitial lung disease	20	7
Lung carcinoma	5	1
Nontuberculous mycobacteriosis	3	
Malignancy disease	2	
Chronic necrotizing pulmonary aspergillosis	1	
Sepsis	1	
Nonspecific findings	5	1

^∗^Some cases were comorbid.

## Data Availability

All the cases' background and characteristic data used to support the findings of this study are available from the corresponding author upon request. The number of new infections in Machida City was based on data published on the Machida City government website (https://www.city.machida.tokyo.jp/iryo/hokenjo/health_crisis_management/number_of_infected.html).
